# Old Maids: Aging and Its Impact on Microglia Function

**DOI:** 10.3390/ijms18040769

**Published:** 2017-04-05

**Authors:** Edward C. Koellhoffer, Louise D. McCullough, Rodney M. Ritzel

**Affiliations:** 1 McGovern Medical School at UTHealth Houston, Houston, TX 77030, USA; Edward.C.Koellhoffer@uth.tmc.edu; 2 Center for Shock, Trauma and Anesthesiology Research (STAR) and Department of Anesthesiology, University of Maryland School of Medicine, Baltimore, MD 21201, USA; rritzel@anes.umm.edu

**Keywords:** aging, microglia, inflammation, dysregulation, senescence

## Abstract

Microglia are highly active and vigilant housekeepers of the central nervous system that function to promote neuronal growth and activity. With advanced age, however, dysregulated inflammatory signaling and defects in phagocytosis impede their ability to perform the most essential of homeostatic functions, including immune surveillance and debris clearance. Microglial activation is one of the hallmarks of the aging brain and coincides with age-related neurodegeneration and cognitive decline. Age-associated microglial dysfunction leads to cellular senescence and can profoundly alter the response to sterile injuries and immune diseases, often resulting in maladaptive responses, chronic inflammation, and worsened outcomes after injury. Our knowledge of microglia aging and the factors that regulate age-related microglial dysfunction remain limited, as the majority of pre-clinical studies are performed in young animals, and human brain samples are difficult to obtain quickly post-mortem or in large numbers. This review outlines the impact of normal aging on microglial function, highlights the potential mechanisms underlying age-related changes in microglia, and discusses how aging can shape the recovery process following injury.

## 1. Introduction and Overview

The U.S. population is aging at a historic pace. The population of those over 65 years is expected to double over the next three decades, and those aged 80 and older are projected to triple in number [1]. By 2050, the global life expectancy is expected to increase by nearly eight years. However, the quality of life of the estimated ~17% of the world’s population soon to be over 65 years will not necessarily be any better due to the increasing burden of age-related diseases such as stroke and other neurodegenerative diseases. The rate of dementia will increase from 9.4% to 23.5% by the mid-century, and the number of people living with Alzheimer’s disease in the U.S. is expected to grow by nearly 10 million [2]. By 2030, nearly 4% of the U.S. population is projected to have had a stroke, straining our already limited resources and health infrastructure [3]. Our scientific understanding of normal aging processes is incomplete and the mechanisms leading to age-related disability with the advent of age-mitigating/rejuvenation therapies needs further exploration.

**Figure 1:**
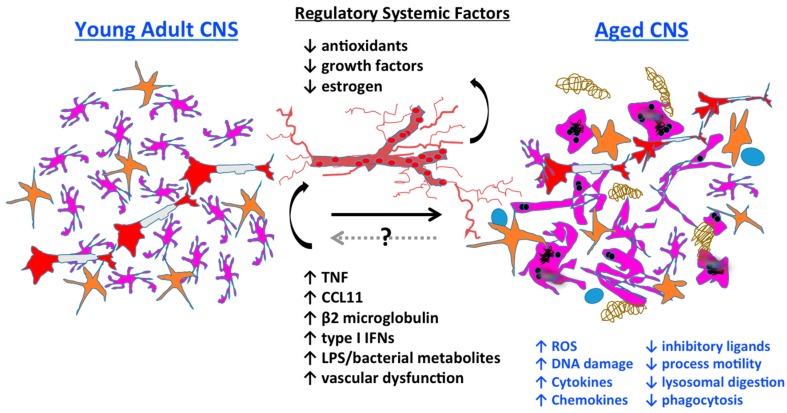
The impact of aging on microglia function and its systemic regulation. Young microglia (in pink) gradually transition from a ramified morphological state to a deramified, spheroid formation with abnormal processes with chronological age. Several cytoplasmic features are hallmarks of microglial senescence including increased granule formation, autofluorescent pigments such as lipofuscin, and process fragmentation. Age-related neuronal loss (in red) reduces the overall level of immunoinhibitory molecules (e.g., CD200, CX3CL1) required to maintain microglia in a quiescent state. Basal increases in inflammatory signaling are associated with enhanced reactive oxygen species (ROS) production which results in the generation of free radicals, lipid peroxidation, and DNA damage. This positive feedback loop is further compounded by defects in lysosomal digestion and autophagy, resulting in the potentially toxic buildup of indigestible material. Concurrent reductions in process motility and phagocytic activity lead to decreased immune surveillance and debris clearance, resulting in plaque formation (in brown). In turn, microglia activation triggers astrocyte activation (in orange) and promotes the recruitment of T cells (in blue) into the aging brain. These pathological features of microglial aging are highly influenced by the systemic environment. Diminished (↓) levels of circulating anti-aging factors in conjunction with increased (↑) concentrations of pro-aging factors are critical drivers of microglial senescence. For example, diminished estrogen levels in older (menopausal) females are associated with elevated expression of macrophage-associated genes in the brain. Therapeutic interventions intended to increase anti-aging factors and decrease pro-aging factors appear to be able to halt or delay microglia aging, enhance neurogenesis, and improve cognitive function.

The effects of aging on the central nervous system (CNS) are widespread, as are systemic changes in peripheral tissues. The importance of communication between the CNS and the periphery is increasingly recognized, and may be mediated by systemic factors, the autonomic nervous system, commensal bacteria (i.e., the microbiome) and/or the neuro-immune axis. Age-related changes in CNS homeostasis are not solely intrinsic in nature, but are mediated through bidirectional communication between the CNS and the systemic environment (Figure 1). Differences in neuronal function have been observed in the CNS with age, but it is becoming increasingly apparent that it is possible to slow, or even reverse, aging by restoring “youthful” peripheral tissue compartments [4,5]. This includes the bone marrow niche that gives rise to the body’s immune system, which can have a beneficial positive feedback effect on distant areas including the CNS. 

**Figure 2:**
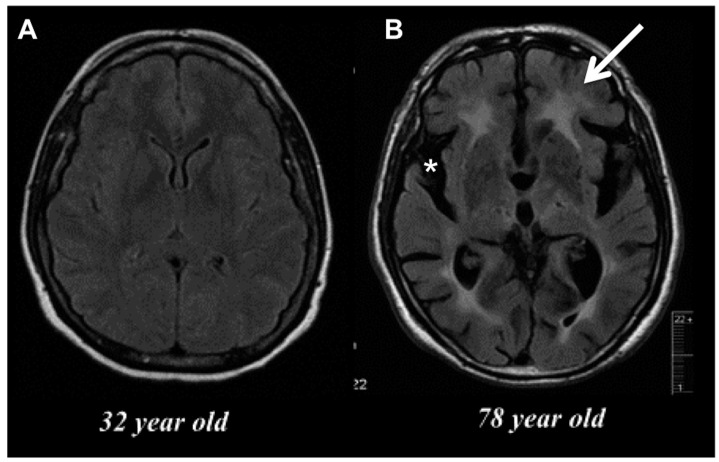
(**A**) Magnetic resonance imaging (MRI) of a normal 32 year old woman. There is no evidence of atrophy or white matter disease; (**B**) MRI of a 78 year old woman with mild cognitive impairment. There is considerable frontal temporal atrophy as seen by an enlarged Sylvian fissure (asterisks) and white matter disease (white arrow).

Inflammation is viewed as a central driver of aging and/or age-related dysfunction. The term “inflamm-aging” was coined to describe the ever subtle but gradual increase in inflammatory signaling with age [6,7]. Although inflamm-aging is primarily macrophage-driven, the accrued effects of this are widespread, affecting nearly all cells at either the intrinsic or extrinsic level—to an extent that fundamentally alters normal physiological behavior—as evidenced by the overall age-related decline in normal function. Characteristics of the aged brain such as gray matter loss and cortical thinning, shrinkage in hippocampal volume, deficits in learning and memory, and decreased remyelination (see Figure 2) are all processes that have been empirically proven to involve inflammation, the severity of which likely depends on the level of degeneration and how it is modulated.

No cell is protected from the detrimental effects of aging, and this includes the primary immune cell of the CNS, the resident tissue macrophages known as microglia. These cells represent 5%–15% of all brain cells, and are considered to be the housemaids of the CNS, providing nourishment and support to neighboring neurons, clearing debris, and being the first responders to foreign stimuli [8]. Like their neuronal counterparts, microglia are believed to be post-mitotic and long-lived, with minimal, if any, turnover. Although recent depletion studies imply the existence of latent microglia progenitors, it is not clear what role this proposed population of cells may have in replenishing microglia populations under normal homeostatic conditions across the lifespan [9]. Thus, these cells may still be viewed as especially vulnerable to the cumulative effects of aging, and thus poised to negatively impact the neurovascular niche as a result of a compromised ability to perform essential ‘house-keeping’ functions. While the role of aging on circulating macrophages and other lymphoid-associated myeloid cells has received significant attention in recent years, our understanding of the age-related changes in the function of CNS-resident microglia is less clear. This review highlights current findings and concepts on the effects of aging on microglia and stresses their potential contribution to inflamm-aging and age-related stress.

## 2. Age-Related Changes in Microglia Phenotype

Aged microglia exhibit increased soma volume, a retraction in processes, and a loss in uniform tissue distribution [10]. Moreover, microglial process speed is significantly slower with age in healthy and injured animals, resulting in less active tissue sampling and impaired synaptic contact [11]. These age-related abnormalities in cytoplasmic structure, deramification, and process fragmentation were collectively termed “microglial dystrophy”, and are more indicative of a senescent rather than classical activation phenotype [12,13]. The newly coined ‘dark’ microglia phenotype defined by condensed electron-dense cytoplasm and nucleoplasm, nuclear chromatin remodeling, and high levels of synaptic stripping activity and oxidative stress applies not only to microglia populations associated with pathological states such as chronic stress and Alzheimer’s disease, but to the microglia that are observed in normal aging [14]. While these alterations at the ultrastructural level are only now beginning to be described, it has been well known that aged microglia are highly granular and atypically dark in appearance in immunohistological preparations. Defects in lysosomal digestion can result in the progressive accumulation of indigestible material largely composed of lipofuscin and other autofluorescent pigments [15,16]. Researchers studying protein expression in aged microglia using immunofluorescence or flow cytometry techniques are likely familiar with the high degree of autofluorescence in these cells. Age-related microglia-associated autofluorescence is often viewed as a technical nuisance as it is difficult to differentiate changes in the normal CNS or those due to severe injury. The accumulation of lipofuscin and other non-degradable autofluorescent byproducts is believed to be due to impairment in disposal mechanisms and has also been implicated in several neurodegenerative diseases including Alzheimer’s disease [17,18]. 

## 3. Strategies to Investigate Functional Characteristics of Microglia

**Figure 3:**
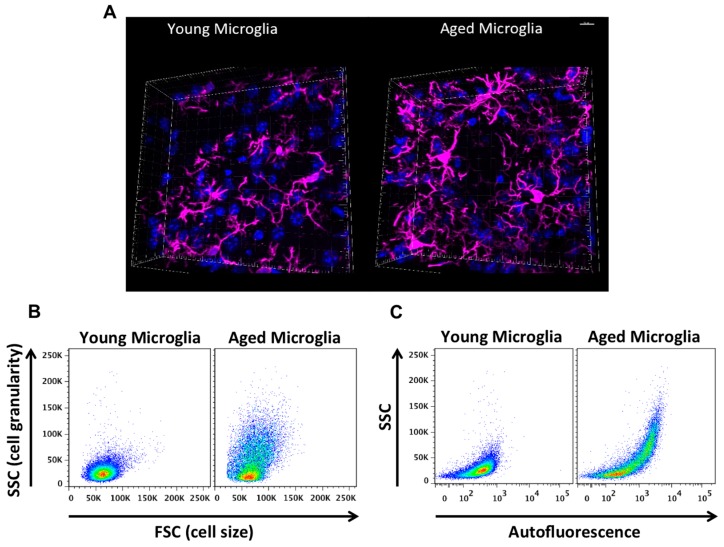
Age-related microglial dystrophy. Confocal microscopy images of DAPI-counterstained (blue) Iba1-positive cortical microglia (pink) highlight the enlarged soma and abnormal, twisted cytoplasmic processes of aged microglia (**A**); Flow cytometry preparation of CD45^int^CD11b^+^Ly6C^−^ microglia demonstrate a significant increase in cellular granularity and size with age (**B**). A population of side scatter-high aged microglia exhibits high levels of autofluorescence in the fluorescein isothiocyanate (FITC) channel compared to their younger counterparts and is indicative of lysosomal dysfunction (**C**).

Not all aged microglia exhibit an “aged” phenotype, and it is unclear whether autofluorescence is confined to dystrophic (i.e., dysfunctional) microglia populations in select regions or if it is a more widespread phenomena, as it is currently difficult to isolate these cells and evaluate their function relative to their non-autofluorescent counterparts. Using flow cytometry, our lab has recently identified a significant population of side scatter-high microglia in the aged brain that exhibit a surprising level of both granular content and autofluorescence background [19]. These cells display functional abnormalities when compared to young microglia and more importantly, to side scatter-low microglia that co-exist alongside them in the aged CNS (see Figure 3). These functional abnormalities include higher production of reactive oxygen species (ROS) and pro-inflammatory cytokines, increased mitochondrial content, and poor phagocytic ability—all features of a senescent or dystrophic macrophage phenotype. However, if the proper precautions are not taken, aged microglia can appear to stain positive for nearly any antibody or fluorescent label. Because of this artifact, the high level of background exhibited by aged microglia requires the use of fluorescence-minus one (FMO) or isotype controls specifically using aged brains and subsequently gating on aged microglia to determine the true background level—which in studies from our laboratory appear to be greater than that of any other immune cell in the body. Other methods of masking or quenching autofluorescence such as Sudan Black B have been developed and are often employed during histological preparations [20]. Given the clinical ramifications of lipofuscin accumulation in both neurons and glia, future efforts to develop brain-imaging techniques which can exploit the naturally occurring level of autofluorescence (i.e., lipofuscin-like material) in the CNS may prove highly useful in predicting or diagnosing neurological disease states in humans. 

Despite several reports demonstrating prolonged maintenance of aged microglia in vitro [21,22], for many, the long-term culture of microglia isolated from aged brains can be technically challenging if not impossible. These methods are invaluable to the study of intrinsic age-related changes in microglia function. One alternative approach is the immediate ex vivo functional assessment of freshly harvested cells. Although some activation is induced during the mechanical/enzymatic digestion procedure via crude extraction from their native microenvironment (e.g., loss of contact-inhibitory signaling), this confounder is seen by many to be unavoidable and perhaps necessary in order to understand their functional activity. Ex vivo functional testing is advantageous for aging studies because it allows the investigator to probe for intrinsic microglia activity in a very acute time window (within hours), obtaining as close to their presumed in vivo functional identity as possible with minimal artifact introduced by standard cell culture systems. Indeed, a recent report suggests that neonatal microglia undergo dramatic ‘age-like’ changes in as short of time from 2 days in vitro to 16 days in vitro [23]. Nonetheless, in vitro approaches are currently indispensable and the advent of more efficient long-term culture methods will hopefully allow researchers to address many important questions that ex vivo testing is not well suited for. 

Another issue regarding the activation status of microglia is the suitability of the existing nomenclature and its application to age-related microglial phenotypes. The long-held M1/M2 convention for describing macrophage polarization may be more applicable to in vitro systems than for far more complex in vivo environments, as mixed phenotypes are commonly seen [24]. The M1/M2 in vitro paradigm, originally premised on infection studies, attempts to explain the predisposition for peripheral macrophages to respond as either “inflammatory” (M1) or “reparative” (M2) subsets depending on their exposure to the cytokine byproducts of polarized T cell subsets (Th1: IFNγ or Th2: IL-4). Subsequent transcriptomic profiling has demonstrated significant differences between bone marrow-derived monocyte populations and CNS-resident microglia, which may be due in part to the age (high vs. low turnover) and environment (circulation vs. brain) of the cells being profiled, and any interactions between the two. An expert analysis of this controversy was recently discussed by Ransohoff [25]. Among the many salient points expressed was the lack of predicted transcriptional organization found between polarization states induced in several disease models as demonstrated by ex vivo expression profiling of microglia, indicating that microglial reactivity is multifactorial and injury-specific, and thus, unlikely even to fall along a linear continuum. The application of M1/M2 markers for the in vivo description of microglia activation states is inadequate in defining the injury-resolving capacity of these cells. Thus, it would seem that attempting to classify the pro-inflammatory phenotype of aged microglia as M1 may be too simplistic in that it ignores the adaptive requirement of these cells to respond to the demands of a changing microenvironment over the lifespan [26]. In recent years, the senescence-associated secretory phenotype, or SASP, has been utilized to more accurately describe aged senescent cells, however these criteria may vary depending on cell type, especially as not all aged cells are senescent per se [27,28]. Aged microglia are likely to exhibit many of the same phenotypic features as other aged post-mitotic tissue-resident macrophages. Although SASP criteria have yet to be established specifically for microglia, emerging studies suggest a framework for one will emerge in the next few years. 

### 3.1. Reactive Oxygen Species-Mediated Damage in the Aging Brain

**Figure 4:**
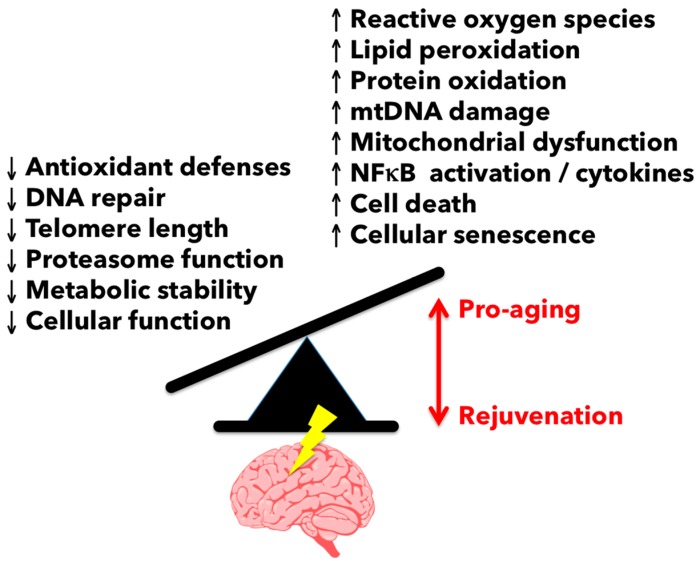
Imbalance between oxidative stress and antioxidant defenses in the aging brain. Oxidative stress arises when there is an excess of free radicals over antioxidant defenses. This imbalance leads to an inability to detoxify the reactive intermediates and results in oxidative damage of genes and proteins. Oxidative stress is a consequence of the aging process and is involved in many diseases such as Alzheimer’s disease, stroke, and atherosclerosis. Stress-activated pathways impact gene expression and alter the normal function of cells, often resulting in respiratory chain dysfunction, altered proteostasis, telomere shortening, apoptosis, and cellular senescence.

One of the most profound changes that occurs with aging is the gradual increase in reactive oxygen species (ROS) generation. Indeed, glial cell activation and elevated oxidative stress burden are hallmarks of CNS aging and manifest during the course of many if not all neurodegenerative diseases (Figure 4). As the predominant myeloid cell in the nervous system, microglia are the main source of oxidation products and inflammatory mediators during aging. Elevated microglia ROS production (e.g., superoxide anion, hydroxyl radical, and lipid hydroperoxides) can impose a hazard to nearby neurons either through direct release (i.e., neurotoxicity) or via second messenger signaling pathways such as PKC, MAPK, and NFκB activation which serve to intensify the pro-inflammatory response [29]. The importance of ROS to age-related neuropathology is evidenced in its key capacity to mediate the detrimental effects of amyloid β (Aβ) and lipopolysaccharide (LPS)-induced CNS injury [30,31]. LPS induces the generation of ROS from the actively respiring mitochondria as well as NADPH oxidase (NOX). Superoxide production via NOX has been shown to be the main contributor of ROS in several age-related neurodegenerative diseases and is linked to the classical activation of microglia [32]. Ansari and Scheff reported an inverse correlation between NOX activity and cognitive impairment, in which higher NOX activity was associated with worse cognitive performance in individuals of all stages of Alzheimer’s disease [33]. Indeed, NOX2-deficiency has been shown to reduce oxidative stress, leading to improved cerebrovascular function and behavior in a mouse model of Alzheimer’s disease. For example, chronic treatment of apocynin, a NOX inhibitor, reduced plaque size and microglia number in hAPP(751) (SL) mice [34].

One of the hallmarks of Alzheimer’s disease is cerebral amyloid angiopathy (CAA), which is characterized by the deposition of Aβ within the walls of cerebral arterioles. Treatment with apocynin and tempol, a non-specific ROS scavenger, attenuated ROS production and improved cerebrovascular function in aged Tg2576 mice [35]. Treated mice exhibited a reduction in CAA formation and CAA-related microhemorrhages, indicating that NADPH oxidase-derived ROS are a key contributor to CAA formation and associated vascular dysfunction. Together, these findings suggest that the age-related increase in microglial ROS production has widespread effects on the neurovascular niche and may be a key accelerant of neurodegenerative disease and cognitive impairment.

Microglia are thought to be the main mediator of ROS-induced neuronal injury and several studies have demonstrated that NADPH oxidase-deficient primary microglia exhibit blunted levels of intracellular ROS, extracellular superoxide, TNF expression, and neurotoxicity following LPS stimulation in vitro [31,36]. NOX may even play a critical role in the development of chronic inflammation as previous work has shown that NOX2-deficient mice exhibit less dopaminergic neurodegeneration in the substantia nigra 10 months after a single systemic LPS injection compared to NOX2^+/+^ controls [36]. It is possible that loss of NOX function attenuates ROS production by the infiltrating neutrophils and monocyte-derivatives in the hours and days following injury. Interestingly though, microglia from NOX2-deficient mice fail to show any increase in activation morphology as early as 1 h following injection, implying that it is the early wave of ROS production that determines the severity of disease course.

As noted above, both NOX and ROS levels increase in the CNS with normal aging and following injury. ROS production and lipid peroxidation is significantly elevated in older mice after contusion spinal cord injury compared to young controls [37]. NOX2 expression was greater in ROS-producing microglia/macrophages in the lesion site of older mice. Interestingly, aging is also associated with a loss of free radical scavenging mechanisms. Antioxidant defenses have been shown to be attenuated in aged microglia, as evidenced by reduced cellular levels of glutathione and dysregulation of heat shock proteins such as heme-oxygenase 1 [38,39]. The potential for antioxidant therapy to improve microglia aging and, in turn, brain aging has recently been reviewed [40]. As of yet, it is still unclear whether worsening injury with age is primarily a result of exacerbated or chronic microglial production of ROS or if aging neurons are just more susceptible to ROS-mediated damage.

### 3.2. Neuronal–Glial Interactions and Immunoinhibitory Signaling in the Aging Brain

In addition to intrinsic age-related stress, microglia are highly responsive to environmental stimuli throughout the lifespan, including extrinsic immunological stressors. As guardians and primary caretakers of the more vulnerable neuronal populations, the manner in which microglia respond to these stressors is critical for normal neuronal function. Activated microglia exhibit augmented production of inflammatory cytokines, ROS, and metabolic byproducts known to be neurotoxic. Thus, given their complex array of activation-sensing receptors and complementary inhibitory receptors, microglia are tightly regulated to deliver calibrated responses to any given stimulus across space (gradient effects) and time (from pathogenesis to resolution phase). To avoid over-activation and any resultant bystander damage, the requirement for microglial inhibitory receptors is essential to not only prevent the generation of unwanted inflammation, but to also ramp down injury-driven inflammatory responses once they are largely resolved. However, with age, the ability of these inhibitory receptors to maintain microglial quiescence is impaired, in part due to reductions in the expression of their cognate ligands. 

### 3.3. CD200/CD200R1

The CD200-CD200R1 immunoinhibitory signaling axis in the CNS is comprised of gray matter neurons that ubiquitously express CD200 both stably on their surface and in secreted form, and microglia/macrophages which express the receptor for this ligand, CD200R1. Microglia/macrophage immune responsiveness is believed to be constitutively down-regulated under normal conditions via direct interactions with neighboring neurons, leading to microglial quiescence. Interestingly, young adult mice that are deficient in CD200, exhibit many features seen in normal aging mice such as basal increases in microglia activation, T cell infiltration, blood–brain barrier permeability, impaired long-term potentiation (LTP), and exacerbated responses to injury and disease [19,41,42,43] implying that CD200 levels may naturally diminish with normal aging [44]. Concomitant increases in transcriptional expression of pro-inflammatory genes and decreases in anti-inflammatory genes such as *CD200* have since been demonstrated in the substantia nigra and hippocampus of older animals [45,46], leading one to speculate that the lack of microglia inhibition leads to greater inflammation which is detrimental to learning and memory. 

CD200 expression is decreased in the brain of Alzheimer’s patients [47] and Aβ-challenged mice [48]. Subsequent in vivo studies demonstrated that intrahippcampal administration of CD200 fusion protein decreased microglial activation and decreased LTP deficits in both aged and LPS-treated rats [49]. Activation of CD200R1 by CD200 fusion protein inhibited Aβ-induced increases in IL-1β, TNF, CD40, and CD68 [50]. Moreover, Aβ-induced deficits in LTP were attenuated by CD200 fusion protein in hippocampal slice culture. Consistent with these findings, the delivery of an adeno-associated virus expressing CD200 into the hippocampus of APP mice for a period of 6 months restored neurogenesis and reduced diffuse plaques in 12-month-old mice [51]. Additionally, the authors found that in vitro stimulation of microglial CD200R1 promoted neuronal growth and despite being anti-inflammatory, resulted in greater Aβ internalization. This is in contrast to a new report that has shown microglia from CD200-deficient mice exhibit increased lysosomal and phagocytic activity in response to Aβ challenge [52]. This effect was mediated in part by mTOR inhibition and implies that CD200 normally functions to suppress immune functions such as phagocytosis. Alternatively, it is possible that the chronic loss of functional CD200 in these knockout models over the lifespan could prime microglia to respond in a manner that is not consistent or comparable to microglia from otherwise normal, healthy wildtype mice. As is the delicate balance of any regulatory system, the potential to increase inhibitory signaling may be offset by the adaptive requirement for activation (e.g., injury-sensing/stimulus recognition, migration to injury sites, debris clearance and phenotype switching). Thus, it is not yet clear whether pro-inflammatory-induced activation of microglia is required to drive phagocytic activity and a return to homeostasis or if chronic inflammation impedes normal function as appears to be the case in the aging brain.

### 3.4. CX3CL1/CX3CR1

The chemokine fractalkine (CX3CL1) which is expressed on neurons in membrane-bound form or secreted by neurons functions similarly to CD200, suppressing activation by binding to its receptor, CX3CR1, expressed on microglia. This interaction is important for downregulating microglial activation and maintaining CNS homeostasis [53,54]. Fractalkine signaling is similarly impaired with normal aging, following LPS challenge, and in APP(swe) transgenic mice, as expression levels for both the ligand and the receptors have been shown to be significantly decreased and inversely associated with inflammatory activity [54,55,56,57]. The age-related loss of fractalkine ligand in the rodent hippocampus is associated with decreased neurogenesis; however, the survival and proliferation of neuronal progenitor cells was restored by exogenous fractalkine, an effect that was not seen in young animals suggesting that this was an age-dependent mechanism [58]. Protracted downregulation of the fractalkine signaling pathway is associated with delayed recovery from sickness behavior, elevated IL-1β levels, and decreased TGFβ production in the aged brain [59]. However predictable the outcome of fractalkine receptor activation on microglia might seem, several reports highlight the complex nature of this immune inhibitory signaling. For example, intrahippocampal injection of Aβ fibrils was found to upregulate CX3CR1 expression on activated microglia and increase synaptic dysfunction and cognitive impairment [60]. It is not surprising that CX3CR1 expression was enhanced by Aβ stimulation, as many immune inhibitory receptors are known to upregulated in classically activated microglia/macrophages in an effort to counteract the pro-inflammatory state. Both CX3CL1- and CX3CR1-deficiency have been shown to reduce Aβ deposition in APPPS1 mice, and an increase in microglial p38MAPK activation and cytokine production [61,62]. In other studies, CX3CR1-deficiency resulted in worsened neuronal and memory deficits in hAPP mice independent of plaque load [63]. Despite the reduction in Aβ plaque deposition, CX3CR1-deficient mice exhibited exacerbated Tau pathology, an effect that was subsequently shown to be suppressed by fractalkine overexpression [64]. Together, these findings suggest that a delicate balance of activating and immunoinhibitory signaling is likely required to perform the full spectrum of function required to maintain homeostasis in the aged CNS environment.

### 3.5. Phagocytosis in the Aging Brain

Debris clearance is an essential role of microglia. Normal aging has significant effects on endocytic pathways, including the phagocytic uptake of debris. Transcriptional analysis of acutely isolated microglia from APPswe/PS1dE9 Alzheimer’s disease (AD) mice reveal diminished expression of genes associated with phagocytosis [65]. At the functional level, young microglia (1 month old mice) internalize ~50% more Aβ42 than aged microglia (15 month old mice), demonstrating an age-related decrease in phagocytic behavior beginning at birth (<8 days old mice) [38]. Data from our laboratory has shown age-related deficits in the phagocytosis of physiological (Aβ) and non-physiological (latex beads) cargo not only at baseline, but also following ex vivo stimulation with PMA/ionomycin [19]. While aging negatively affects the ability of microglia to phagocytose Aβ, it does not appear to limit their ability to adhere to amyloid plaques or in vitro fibrillized Aβ [66]. Moreover, aging did not affect the functional uptake of bacterial bioparticles, and others have reported that aged microglia exhibit greater uptake of quantum dots [67], implying that aging may differentially affect phagocytic pathways at various stages (adherence, internalization, digestion, etc.) or with different substrates. In an interesting study by Hendrick in 2014, the authors showed that while aging enhanced microglial capacity for myelin phagocytosis, it simultaneously reduced myelin’s susceptibility for uptake, suggesting that age-related phagocytic impairment may be mediated both by intrinsic and extrinsic factors, depending on the nature of the substrate [68]. 

Inhibitory “don’t eat me” signals that prevent host attack such as CD47 have also been shown to prevent microglia phagocytosis of healthy cells via activation of the immune inhibitory receptor signal regulatory protein-α [69], although it is unclear what role, if any, molecules such as these have in aging. The ability of a given phagocytic substrate to induce microglia activation either through toxicity or via receptor-mediated signaling may in turn alter the phagocytic potential of that cell [70]. For example, aging decreases microglia and monocyte uptake of α-synuclein oligomers and is associated with increased TNF secretion [71]. The age-related increase in microglia cytokine production, specifically TNF family members, has been demonstrated numerous times and is a hallmark of CNS aging. However, the nature of the relationship between increased TNF production and microglia phagocytosis is one that warrants greater examination, as several studies support an inverse relationship with age. For example, TGFβ-induced phagocytosis is abolished in aged microglia compared to their younger counterparts, indicating that receptor-signaling pathways are significantly altered with age and may underlie endocytic impairment [22,72]. The interactions between cytokine signaling and phagocytosis have proven to be highly complex and provocative, as newly emerging data suggests that microglial phagocytosis and plaque clearance may be suppressed as a result of an overproduction of anti-inflammatory molecules such as IL-10 and arginase-1, rather than mediated by pro-inflammatory dysfunction [73,74,75]. While our understanding of age-related changes in microglial phagocytosis and changes in the expression of scavenger receptors (i.e., CD36) is in its infancy, the development of novel drugs that are capable of directly modulating phagocytic activity and reducing plaque load is becoming a realistic goal. 

### 3.6. Microglial Depletion and Implications to Aging

Just as conditional genetic targeting approaches have advanced our understanding of the molecular mechanisms underlying microglial activation, recent pharmacological and genetic microglia depletion studies have aided our understanding of the net effect of these cells on aging and age-related cognitive decline. These innovative strategies take advantage of several known aspects of microglial identity and requirements for survival. For instance, colony-stimulating factor 1 receptor (CSF1R) is essential for the growth and survival of microglia and other monocyte-derived cells. CSF1R blockade with PLX5622 eliminates microglia for sustained periods of time, allowing for the long-term investigation of microglia in neurodegenerative disease models [76]. PLX5622-induced depletion of microglia prevented their association with plaques, but did not alter amyloid-β levels or plaque load. Importantly, this strategy was able to reduce overall neuroinflammation and attenuated contextual memory deficits in 10-month-old 3xTg-AD mice, without having any overt effect on behavior or cognition in normal adult wild type mice. It is unclear whether microglial depletion in wild-type aged animals has the potential to have a positive cognitive benefit. Administration of the CSF1R inhibitor PLX3397 improved functional recovery and spared neuronal loss, in part by reducing chronic inflammation in 5–8 month old mice following a hippocampal lesion induced by diphtheria toxin exposure [77]. This strategy, similar to what was seen with other CSF1R treatments, was accompanied by an increase in dendritic spine density, suggesting these cells have critical roles in sculpting synapses even after development. PLX5622 also prevented whole-brain irradiation-induced memory deficits in young mice, in part by limiting microglia proliferation and monocyte infiltration [78,79]. These studies have enhanced our knowledge of both the homeostatic role of microglia and their functional contribution to disease pathology. Insights into the global effects of dystrophic microglia in normal aging using similar depletion approaches could prove extremely useful to our understanding of the contribution of microglial senescence to age-related cognitive decline.

### 3.7. Systemic Regulation of Microglia Aging

Although it is true that the CNS is largely protected from the systemic environment, it is not impermeable to it. Indeed, systemic administration of LPS has been found to induce microglia activation, neurodegeneration, and sickness behavior [80]. Thus, it is evident that the brain is influenced by changes in peripheral homeostasis. Although the brain is protected by the blood–brain barrier, there are several conduits through which systemic messages reach the CNS, including the vascular and lymphatic networks, and via the choroid plexus and cerebrospinal fluid. These anatomical interfaces, which likely convey vital information under healthy conditions, may also predispose the brain to the detrimental effects of systemic aging. Work by Baruch et al demonstrated that aging induces a type I interferon (IFN)-dependent gene expression profile in the choroid plexus [81]. Interestingly, blocking IFN-I signaling in the aged brain down-regulated IFN-I-dependent gene expression in the choroid plexus, restored hippocampal neurogenesis, improved cognitive function, and partially reversed age-related glial activation. These findings suggest that age-related microglial dystrophy (i.e., senescence) can be reversed by external modulation. Other blood–brain borders may similarly affect microglia function with age in a location-dependent fashion. Regional heterogeneity in microglia function and differences in immune vigilance are likely to become exacerbated with age as neurovascular function and blood vessel integrity has been reported to be selectively compromised in the hippocampus and frontoparietal cortex [82,83,84]. Pioneering studies using heterochronic parabionts and plasmapheresis support a strong link between the systemic environment and brain aging. Systemic immune factors such as CCL11 and β2-microglobulin are elevated in an age-dependent manner in the plasma and hippocampus, and impair neurogenesis and cognitive function [85,86]. Systemic exposure to either molecule induced similar deficits in learning and memory that were reversed by antibody blockade. These studies convincingly demonstrate the important link between the brain and the periphery. How this bi-directional communication occurs is a very active and novel area of research, as drug and antibody delivery is much easier in the periphery due to poor blood–brain penetrance. Manipulating systemic factors to influence brain inflammation in very appealing for therapeutic development. 

One example of bidirectional communication is illustrated by studies examining the gut–brain axis. Recent work has demonstrated a modulatory effect of gut microbiota on microglia function [87]. Germ-free mice exhibited altered microglia morphology and impaired responses to LPS stimulation and viral infection [88]. Similar changes in microglial phenotype were seen following partial ablation of microbiota by oral antibiotics that were normalized following recolonization with microbiota from specific-pathogen free mice. Mice deficient in the short-chain fatty acid (SCFA) receptor FFAR2 had similar deficits in microglia function as did germ-free mice, suggesting that microbiota-derived SCFA exert systemic control over microglial development and homeostasis. While compositional shifts in microbiome complexity have been shown to occur with age, the significance of these changes and their effect on microglial behavior remains to be seen. Nevertheless, these studies highlight a critical role for the systemic environment in regulating microglia activity and even suggest that brain aging may be mitigated by healthy lifestyle choices and dietary manipulations [89,90,91,92].

## 4. The Role of Aging on the Microglial Response to Brain Injury and Disease

As reviewed above, microglia undergo drastic phenotypic changes with age. These molecular and cellular changes are many, and their summation leads to a dysregulated microglia phenotype. Additionally, these phenotypic changes may be influenced by the changes in the periphery, including microbiome changes [88], age-associated perfusion deficits within the brain itself [93], and even diet [94]. Aging is, no doubt, a complex process and takes its toll on microglia phenotype and function in homeostasis. What is also crucial to consider are the effects of aged microglia in the context of injury and disease in both humans and the animal models we use to study them. Below, we review the effects of age-associated changes in microglia function in several neurological diseases.

### 4.1. Aging Exacerbates Lipopolysaccharide-Induced Pro-Inflammatory Microglial Response

As animals and humans age, there are notable changes in cognition and reaction times even in healthy subjects, and may be an unavoidable consequence of aging [95]. However, studies in animals have shown that age-related cognitive decline is correlated with higher levels of pro-inflammatory cytokines produced by microglia [19] and that the aging brain is sensitized to immune challenges [96]. Aged individuals are more susceptible to delirium when ill with a peripheral infection, stress, or following surgery, recently reviewed elsewhere [97,98,99,100]. This is also observed in animal models (see Table 1). These symptoms are associated with activation of the peripheral innate immune system, and in turn leads to exacerbated inflammatory responses in the aged brain [101,102,103].

Peripheral infection may be modeled in rodents using intraperitoneal injections of lipopolysaccharide (LPS), which is a component of the cell wall of Gram-negative bacteria and a potent stimulator of Toll-like receptor (TLR) 4 signaling. This model rapidly induces a pro-inflammatory response. Additionally, some labs have begun modeling infection with peripheral injection of *Escherichia coli* to control for weight-dependent dosing, as aged mice are heavier and require larger quantities of LPS [104]. LPS can be injected peripherally and rapidly induces a neuroinflammatory response with associated sickness behaviors in rodents, characterized by lethargy, reduced activity, fever, and social withdrawal [110]. This behavior is an evolutionarily preserved response, as social withdrawal during infection is a protective mechanism to prevent the spread of illness to others. This behavioral response may be, in part, due to the elevated production of cytokines and systemic stress factors that enter the brain parenchyma via the circumventricular organs or indirectly through activation of endothelial cells and nearby perivascular macrophages. While this is a seemingly positive protective mechanism, the CNS of aged animals respond in an exaggerated manner to peripheral LPS injection, with higher immediate expression of pro-inflammatory genes and delayed behavioral recovery [101]. This demonstrates that aged microglia are “primed” to respond more robustly to a pro-inflammatory stimulus. 

While the phenomena of microglia priming with age has been well-established, the molecular mechanisms underlying this phenotype have only recently been investigated. Recently, it was found that high mobility group box 1 (HMGB1) protein levels are elevated in the hippocampi and cerebrospinal fluid (CSF) of aged rats. HMGB1 is secreted by various immune cells including microglia as a danger signal [104] and in turn stimulates cells via the TLR4 and receptor for advanced glycation end-products (RAGE) receptors to activate pro-inflammatory gene expression. Blockade of HMGB1 with a pharmacological inhibitor, Box-A, was able to abrogate the priming phenotype of microglia in aged rats in vivo through reductions of MHCII and TLR4 expression in the hippocampus [104], two notable molecules up-regulated in microglial priming. Treatment with Box-A also improved functional recovery in social exploration tasks in aged animals [104] and enhanced freezing behavior in contextual fear conditioning tests, showing an improvement in cognitive function [104]. Further work examining strategies to reverse the primed phenotype of aged microglia will provide novel targets to reduce dysfunctional neuroinflammatory responses in the elderly after neurological injury.

### 4.2. Aged Microglia Contribute to Enhanced Pathology Following Traumatic Brain Injury (TBI)

Elderly patients are more vulnerable to traumatic brain injury (TBI), with a doubling of TBI incidence every 10 years beginning at the age of 65, mostly related to increased falls [111,112]. Age is an independent predictor for mortality after TBI [113,114] and older patients that survive their injury have reduced functional recovery compared to younger individuals [115,116]. Additionally, adults age 55 years or older suffering a moderate to severe TBI or those 65 or older suffering a mild TBI have an increased risk of developing dementia compared to younger patients [117]. Together, these clinical findings suggest that the brain becomes more sensitive to TBI as we age, and that the pathological consequences of injury are much graver. Many factors contribute to this age-related vulnerability including increased amyloid deposition [118], however, it is not clear whether inflammation drives the exacerbation of injury or if it is secondary or correlative to the increase in neuronal death seen in older individuals.

TBI may be modeled in animals using a variety of methods [119,120,121]. In rodents administered a controlled cortical impact (CCI), microglia are activated and express higher levels of pro-inflammatory genes and lower or undetectable levels of anti-inflammatory genes even months after the initial injury, suggesting an overall shift in microglia phenotype toward a pro-inflammatory status with time following TBI. Aged mice that underwent CCI displayed higher MHCII expression [122] and had higher expression of NADPH oxidase subunits p22^phox^ and gp91^phox^. Aged mice also had a corresponding reduction in the expression of antioxidant enzymes superoxide dismutase 1 (SOD1) and glutathione peroxidase 1 (GPX1) following TBI compared to young mice, indicative of higher ROS production in aged mice [122]. These results corresponded with larger lesion volume and reduced cellular density in the hippocampus and thalamus of aged mice 7 days after CCI [122]. Together, these results suggest that microglia in the aged brain are more detrimental in TBI and may predispose the aged to impairments in phagocytosis and increased accumulation of toxic waste products such as amyloid.

In addition to contributing to the excessive pro-inflammatory status in the aged brain, microglia have been shown to be directly involved in post-TBI recovery, including neurite outgrowth. Nogo receptor 1 (NgR1) signals through the RhoA-ROCK pathway and results in the collapse of the growth cone of neurites [123]. Thus, higher levels of NgR1 are associated with reduced neurite outgrowth. NgR1 was found to be highly expressed in Iba1^+^ microglia in the cortex and basal forebrain beginning at P1 and declined until stable baseline adult levels were achieved by P21 [124]. However, expression levels were increased in mice at 17 months of age [124], suggesting that aged microglia may predispose the aged brain to decreased neurite outgrowth following an acute injury. In young animals with TBI from a stab wound model, the number of Iba1^+^ NgR1^+^ microglia increased relative to sham controls, although total levels of NgR1 measured by Western blot did not increase 7 days post-injury [124]. However, given the different phenotype of microglia in the aging brain, future studies in aged mice will be necessary to confirm the effects of elevated NgR1 in aged microglia following TBI.

It is not difficult for one to imagine the synergistic effects of aging and a prior TBI, as aging itself primes microglia in a similar manner as TBI. Thus, the response to TBI in aged animals is noticeably worse, and the response of repetitive TBI in aged animals is worse still. With the observation of increased incidence of TBI with each decade of life after the age of 65, repetitive TBIs are not only more common, but the consequences of each additional TBI are likely more severe than the previous TBI, leading to progressive deficits and cerebral atrophy. 

### 4.3. Aged Microglia Contribute to Worse Recovery and Functional Outcomes Following Stroke

Age is the leading non-modifiable risk factor for stroke. While preclinical studies in animal models have mostly been performed in young animals, there are many laboratories that are now transitioning their studies into aged animals. Popa-Wagner’s group in particular has demonstrated profound changes in microglia activation after stroke with age [125,126,127]. While more expensive and surgically challenging, this is an increasingly important consideration as the response to stroke differs in young and aged animals [43,128,129]. Perhaps most importantly, aged animals have comorbidities that resemble those seen in clinical populations, including obesity [130] and hypertension. Thus, modeling stroke in aged animals allows for a more accurate modeling of stroke [127]. However, variations in stroke models (e.g., transient vs. permanent occlusion of vessels, time duration of infarction, age of “aged” animals, strain of mice, etc.) make it difficult for laboratories from different groups to compare studies.

Interestingly, despite the high level of cell death that occurs following cerebral ischemia, one study reported that microglia have a more anti-inflammatory phenotype with higher expression of *CD206*, *IL10*, *YM1/2*, *TGFB*, *ARG1*, and *CCL22* in the first 3 to 5 days following stroke, with a transition to higher expression of pro-inflammatory genes *CD16*, *CD32*, and *NOS2* at 7 and 14 days post-stroke [131]. While this study examined RNA expression from an entire stroke hemisphere, the phenotype of microglia following stroke is likely influenced by their relative location to the infarcted region where cell death and pro-inflammatory stimuli are abundant. However, these authors found corresponding patterns of expression of CD16/32^+^ Iba^+^ and CD206^+^ Iba1^+^ microglia using immunofluorescence in the peri-infarct region of the stroke consistent with their gene expression findings [131]. The timing of phenotype switching likely depends on the stroke model used, and the brain area assessed. 

Similar findings were also found in aged animals, with higher Iba1^+^ CD206^+^ microglia peaking at 7 days post-stroke, and then transitioning to elevated Iba1^+^ CD16/CD32^+^ microglia at 14 days post-stroke [132]. However, despite this pattern of early anti-inflammatory polarization followed by delayed pro-inflammatory polarization in both young and aged mice, aged mice had reduced anti-inflammatory polarization relative to young animals [132]. The authors also found a positive correlation between the number of Iba1^+^ CD206^+^ microglia and improved cognitive and motor performance, suggesting that the reduced ability of aged microglia to efficiently polarize to an anti-inflammatory phenotype may be responsible for poorer functional recovery following stroke [132]. However, the exact mechanism linking the inflammatory status of microglia to functional outcomes remains to be elucidated.

As noted earlier, microglia of aged animals have reduced cellular motility and phagocytosis, and augmented production of pro-inflammatory cytokines. These baseline differences have implications for microglial activation after stroke. In a distal MCAO (dMCAO) model of stroke, aging increased microglial proliferation in the peri-infarct area [133]. Additionally, while aging was not shown to reduce proliferation of neuroblasts within the subventricular zone, there was an age-related reduction of migration of these neuroblasts to the peri-infarct area [133]. Perhaps the enhanced proliferation of microglia (and astrocytes) in the peri-infarct area of aged animals is detrimental to neuronal repair processes following stroke leading to worse behavioral outcomes.

Interestingly, aging is associated with an accumulation of resident memory CD8 T cells in the brain parenchyma [43]. At baseline, higher numbers of CD8 T cells correlate with reduced pro-inflammatory functions of microglia in the aged brain; however, following tMCAO, these CD8 T cells had increased TNF, IFNγ and CCL2 as determined by intracellular cytokine staining [43]. Together, these results suggest that CD8 T cells within the aging brain modify microglia homeostasis under naïve conditions but may be another source of priming the aging brain and potentiating damage following tMCAO. The role of these resident memory CD8 T cells in the aging brain will need to be evaluated in other disease models to determine their importance and relevance to disease.

It has also become increasingly apparent that inflammatory changes following ischemic stroke are not limited to the CNS but are evident throughout the body, including peripheral immune organs such as the spleen. Following tMCAO, both young and aged mice have elevated gut permeability and translocation of intestinal microbes, but aged mice had prolonged loss of body weight, severe hypothermia, and persistent elevation of plasma IL-6 production at 72 h post-stroke, indicating that aged animals were not able to resolve the infection [128]. While not directly assessed in this study, bacterial byproducts themselves (e.g., LPS) may be elevated in the blood of aged mice following stroke and, given the increased level of blood–brain barrier breakdown after ischemic injury, may directly activate microglia and further potentiate the pro-inflammatory polarization of microglia in the aged brain. Further studies investigating these mechanisms are necessary to understand how peripheral factors could influence microglia polarization and function, both under healthy conditions and in response to injury and disease.

### 4.4. The Role of Aged Microglia in Alzheimer’s Disease

Alzheimer’s disease (AD) is a neurodegenerative disease primarily affecting older individuals and is the most common cause of dementia (Ballard 2011, Braak 1997). The earliest clinical manifestation of AD is memory impairment, and this is usually present at the time of clinical presentation/diagnosis of the disease. Currently available treatments only target the symptoms of AD, as there are no disease-modifying therapies available for the treatment of AD. New therapeutic approaches include vaccines targeted against Aβ and infusions of antibodies targeting Aβ such as bapineuzumab; however, these approaches have failed in clinical trials to improve clinical outcomes [134] despite being well-tolerated by patients [135,136,137]. While these results were disappointing, there is renewed hope in the clinical effectiveness of aducanumab, an antibody that selectively targets aggregated Aβ which appears to show promise in preclinical studies and clinical trials [138]. However, even if these new biological therapeutics slow or even halt the progression of AD, aged patients have other etiologies for cognitive decline beyond amyloid-beta pathology, such as vascular dementia, for which these drugs are likely to be less effective.

The AD brain has many specific pathological findings [139]. These include aggregations of Aβ, hyperphosphorylated tau, neurofibrillary tangles, and glial cell activation. While the exact cause of Alzheimer’s disease pathogenesis remains debatable, it is becoming clear that microglia play a crucial role in disease pathology, which has been recently reviewed elsewhere [140,141,142]. The hippocampus is the area of the brain most densely populated by microglia, and is also one of the brain regions that is affected early in AD and leads to many of the clinical symptoms [39,143,144]. The exact trigger for microglia activation remains unclear, but Aβ itself is capable of directly activating microglia [145,146,147]. Accumulation of extracellular Aβ may be due to impaired phagocytosis of abnormal proteins by aged microglia. Aged microglia have poorer phagocytosis compared to microglia from young mice [19], potentially leading to accumulation of Aβ in the extracellular environment, further formation of Aβ aggregates, and a subsequent further activation of microglia [66,148]. 

The reduced ability of microglia to phagocytose Aβ may be due to a decreased ability of microglia to directly bind and degrade Aβ. One study specifically comparing phagocytosis of Aβ by microglia in young and aging PS1-APP mice found that microglia from 8-month-old PS1-APP mice demonstrated significantly reduced RNA expression of Aβ binding receptors *SRA*, *CD36*, and *RAGE* relative to age-matched wildtype littermates, and levels declined even further by 14-months of age [149]. Furthermore, reduced expression of Aβ degrading enzymes insulysin (*IDE*), neprilysin (*MME*), and matrix metallopeptidase 9 (*MMP9*) was seen by 14-months of age [149]. These findings suggest that in the setting of AD, microglia become increasingly inefficient at their ability to clear Aβ with age, highlighting yet another functional failure of aged microglia.

The inflammatory nature of aged microglia likely also plays a role in AD pathogenesis. Microglia in naïve wildtype aged mice express elevated levels of IL-1β and TNF [19] compared to young microglia, suggesting that the pro-inflammatory “activated” phenotype occurs in the absence of AD or any other pathology and are a hallmark of aging in the CNS. In the setting of AD pathology, microglia PS1-APP mice were found to have elevated RNA expression of *IL1B* and *TNFA* at 8-months of age with further elevation in expression by 14-months [149]. N9 microglia cells treated with TNF were found to have decreased expression of phagocytic receptors SRA and CD36, and in turn also had decreased uptake of Aβ [149]. This suggests that elevation of inflammation from microglia within the aging brain reduces their ability to effectively phagocytose Aβ, thereby contributing to the pathogenesis and/or progression of Alzheimer’s disease.

Recently, experiments have been performed using plasma transfers and parabiosis models to examine the contribution of peripheral cells and circulating factors in AD pathology, as summarized in Table 2.

The most recent study utilized a model of heterochronic parabiosis to explore the possibility that circulating factors in young blood may prevent AD pathology and progression. Heterochronic parabiosis is a model in which a young animal is surgically attached to an aged animal and through anastomoses of the wound healing process come to share a common blood supply. This model has been shown to have significant effects on neurogenesis and identified circulating molecular markers of aging, particularly CCL11 and β2-microglobulin [85,86]. Heterochronic parabiosis with young 2–3-month-old wildtype mice and old 16–20-month-old APP transgenic mice showed that after 5 weeks of shared circulation, no reduction in total Aβ or Aβ-42 was seen in the hippocampus of aged APP heterochronic mice compared to APP isochronic mice [150]. Interestingly, there was no difference in CD68 immunoreactivity (a lysosomal protein enriched in myeloid cells including microglia [153]) in the hippocampi of these mice, suggesting that once pathology is established, the potential for reducing progression or reversing plaque burden may be limited. However, despite the inability of heterochronic parabiosis to delay or reverse disease pathology, APP mice had increased synaptophysin and calbindin immunoreactivity in the hippocampus, suggesting that circulating factors in young blood may at least be able to restore synaptic protein levels despite a lack of reduction in total Aβ or Aβ-42 plaque levels [150]. While behavioral outcomes cannot be assessed in parabionts due to the surgery, these may be more accurately assessed in similar studies using plasma transfer experiments [85,86]. Similar to heterochronic parabionts, APP mice receiving blood from young wildtype donors also demonstrated increased synaptophysin and calbindin immunoreactivity relative to APP mice receiving phosphate-buffered saline (PBS). APP mice receiving plasma from young wildtype donors were shown to have improved performance on Y-maze and contextual fear conditioning tests [150]. Together, these results suggest that factors from young healthy donors may be able to improve functional performance in diseased animals despite the inability to delay or reverse disease progression. Further investigation of potential “rejuvenation factors” in young plasma will be necessary to determine which factors may be responsible for these changes.

## 5. Conclusions

The U.S. population is aging at an alarming rate and the increase in age-related diseases such as stroke, TBI, and Alzheimer’s disease will place an increasing strain on our healthcare system. Chronic age-related increases in inflammation exist in both the periphery and CNS. Microglia, like other long-lived cells, may be especially vulnerable to the detrimental effects of aging, as reflected by changes in their molecular and cellular phenotype, decreased phagocytic potential, and increased production of ROS. These pro-inflammatory and primed microglia play a substantial role in the pathogenesis and progression of age-related neurological diseases and in the exaggerated response to injury and infection seen in the aged. Understanding the mechanisms by which microglia age will enhance the identification and development of novel intervention strategies to reduce the burden of age-related neurological diseases.

## Figures and Tables

**Table 1:** Summary of animal studies comparing young and aged differences in cognitive function following stress. Various models of stress and immune system activation examined in young and aged animals show that aged animals have an exaggerated neuroinflammatory response and prolonged behavioral deficits compared to young animals. (↑ = “elevated,” ↓ = “reduced”)

Stressor	Study	Animals	Age(s)	Sex	Model	Notable Findings
Peripheral infection	[101]	BALB/c mice	Young 3–6 m Aged 20–24 m	Male	Lipopolysaccharide (LPS) i.p. injection	Exaggerated ↑ IL-1β, IL-6, lipid peroxidation in aged brain ↓ social behavior, food intake, weight loss in aged
	[104]	F344XBN rats	Young 3 m Aged 24 m	Male	Live *E. coli* i.p. injection	At baseline: ↑ hippocampal HMGB1 protein, mRNA in aged; ↑ HMGB1 protein in cerebrospinal fluid (CSF) of aged Following i.p. *E. coli* injection: Prolonged ↑ expression of IL-1β, IL-18, TNF in aged; prolonged sucrose anhedonia (depression) and ↓ juvenile social exploration in aged Inhibition of HMGB1: abrogated primed phenotype of aged brain to peripheral *E. coli* injection, restoring behavior to that of young animals
Central innate immune activation	[105]	BALB/c mice	Young 3–4 m Aged 20–22 m	Male	LPS i.c.v. injection	Prolonged ↓ locomotor activity, social behavior, and food intake in aged ↑ cerebellar and hippocampal IL-1β, IL-6, and TNF expression in aged
Surgery	[106]	BALB/c mice	Young 4–6 m Aged 23–25 m	Male	1.5 cm abdominal incision and gentle manipulation of internal organs for 1 min	Anesthetic and analgesics: no effect on hippocampal IL-1β, IL-6 and TNF mRNA expression Surgery: ↑ IL-1β expression in aged hippocampus; locomotor activity unchanged in in young or aged mice
	[107]	C57Bl6/J mice	4 m	Female	0.5 cm abdominal incision	Surgery ↑ anxiety, ↓ special memory
	[108]	C57Bl6/J mice	2 m–8 m	Female	Simple laparotomy	Surgery ↑ total alpha-synuclein and S100β in the cortex, ↓ attention
Stress	[109]	BALB/c mice	Young 3–5 m Aged 22–24 m	Male	30 min restraint stress daily for 4 days	Stress ↑ weight loss, exaggerated ↑ hippocampal and hypothalamic IL-1β mRNA expression in aged; exaggerated ↑ corticosterone in aged Higher hippocampal MHCII mRNA and immunohistochemistry staining in aged mice at baseline, and increased in aged mice following stress

**Table 2:** Summary of studies examining the role of the peripheral immune system on Alzheimer’s disease (AD) pathology. Recent experiments utilizing models of parabiosis and plasma transfers are beginning to address the role of and the extent to which the peripheral immune system and soluble plasma factors may be manipulated in modifying AD pathology and cognition. (↑ = “elevated,” ↓ = “reduced”)

Study	Strain	Age(s)	Model	Duration	Notable Findings
[150]	APP on C57Bl/6 background	Young 2–3 m Aged 16–20 m	Heterochronic parabiosis Aged APP—Young WT Aged APP—Aged APP Aged WT—Aged WT	5 weeks	In the hippocampus: rejuvenation of synaptophysin and calbindin immunoreactivity; no change in total Aβ or Aβ-42 levels; no effect of CD68 immunoreactivity
			Plasma transfer PBS Young plasma	Administration twice weekly for 4 weeks	In the hippocampus: rejuvenation of synaptophysin and calbindin immunoreactivity; no effect of CD68 immunoreactivity Improved memory, spatial learning memory with young plasma transfer
[151]	APPswe/PS1dE9 Tg	Young 3 m Tg 3 m	Heterochronic parabiosis Young Tg—Young WT Age-matched Tg Age-matched WT	6 months	In heterochronic Tg parabionts: ↓ Aβ-40, Aβ-42, total Aβ, and Congo Red plaques in brain ↓ CAA vessel number and area Alleviation of neuronal degeneration and apoptosis
[152]	B6.CD45.1 5XFAD (CD45.2)	4 or 8 m	Parabiosis B6.CD45.1–5XFAD	4 weeks	No recruitment of CD45.1 WT monocytes to brains of 5XFAD parabionts Brain-resident microglia associate with amyloid plaques, not peripheral monocytes
	B6.CD45.1 APP-PS1 (CD45.2)	3.5 m	Parabiosis B6.CD45.1–APP-PS1	9 weeks	No recruitment of CD45.1 WT monocytes to brains of APP-PS1 parabionts

## Abbreviations

AD, Alzheimer’s disease; APP, amyloid precursor protein; ARG1, arginase-1; ATP, adenosine triphosphate; Aβ, amyloid β; CAA, cerebral amyloid angiopathy; CD, cluster of differentiation; CCI, controlled cortical impact; CCL, chemokine (C-C motif) ligand; CSF, cerebrospinal fluid; CSF1R, colony stimulating factor 1 receptor; DNA, deoxyribonucleic acid; FFAR2, free fatty acid receptor 2; FMO, fluorescence minus one; GPX1, glutathione peroxidase 1; HMGB1, high mobility group box 1 protein; Iba1, ionized calcium-binding adapter molecule 1; IFN, interferon; IL, interleukin; iNOS, inducible nitric oxide synthase; LPS, lipopolysaccharide; LTP, long-term potentiation; MAPK, mitogen-activated protein kinase; MHC, major histocompatibility complex; MRI, magnetic resonance imaging; mTOR, mechanistic target of rapamycin; NADPH, nicotinamide adenine dinucleotide phosphate; NFκβ, nuclear factor κ β; NgR1, nogo receptor 1; NOX, NADPH oxidase; PBS, Phosphate-buffered saline; PKC, protein kinase C; RAGE, receptor for advanced glycation end products; RNA, ribonucleic acid; ROS, reactive oxygen species; SCFA, short chain fatty acids; SOD1, superoxide dismutase 1; SRA, scavenger receptor A; TBI, traumatic brain injury; TGFβ, transforming growth factor β 1; TLR4, toll-like receptor 4; tMCAO, transient middle cerebral artery occlusion; TNF, tumor necrosis factor.

## References

[1] He W., Goodkind D., Kowal P.R., U.S. Census Bureau (2015). An Aging World: 2015.

[2] Eshkoor S.A., Hamid T.A., Mun C.Y., Ng C.K. (2015). Mild cognitive impairment and its management in older people. Clin. Interv. Aging.

[3] Ovbiagele B., Goldstein L.B., Higashida R.T., Howard V.J., Johnston S.C., Khavjou O.A., Lackland D.T., Lichtman J.H., Mohl S., Sacco R.L. (2013). Forecasting the future of stroke in the United States: A policy statement from the American Heart Association and American Stroke Association. Stroke.

[4] Wyss-Coray T. (2016). Ageing, neurodegeneration and brain rejuvenation. Nature.

[5] Bouchard J., Villeda S.A. (2015). Aging and brain rejuvenation as systemic events. J. Neurochem..

[6] Salvioli S., Capri M., Valensin S., Tieri P., Monti D., Ottaviani E., Franceschi C. (2006). Inflamm-aging, cytokines and aging: State of the art, new hypotheses on the role of mitochondria and new perspectives from systems biology. Curr. Pharm. Des..

[7] De Martinis M., Franceschi C., Monti D., Ginaldi L. (2005). Inflamm-ageing and lifelong antigenic load as major determinants of ageing rate and longevity. FEBS Lett..

[8] Wake H., Moorhouse A.J., Miyamoto A., Nabekura J. (2013). Microglia: Actively surveying and shaping neuronal circuit structure and function. Trends Neurosci..

[9] Elmore M.R., Najafi A.R., Koike M.A., Dagher N.N., Spangenberg E.E., Rice R.A., Kitazawa M., Matusow B., Nguyen H., West B.L. (2014). Colony-stimulating factor 1 receptor signaling is necessary for microglia viability, unmasking a microglia progenitor cell in the adult brain. Neuron.

[10] Wong W.T. (2013). Microglial aging in the healthy CNS: Phenotypes, drivers, and rejuvenation. Front. Cell. Neurosci..

[11] Hefendehl J.K., Neher J.J., Suhs R.B., Kohsaka S., Skodras A., Jucker M. (2014). Homeostatic and injury-induced microglia behavior in the aging brain. Aging Cell.

[12] Streit W.J., Braak H., Xue Q.S., Bechmann I. (2009). Dystrophic (senescent) rather than activated microglial cells are associated with tau pathology and likely precede neurodegeneration in Alzheimer’s disease. Acta Neuropathol..

[13] Streit W.J., Sammons N.W., Kuhns A.J., Sparks D.L. (2004). Dystrophic microglia in the aging human brain. Glia.

[14] Bisht K., Sharma K.P., Lecours C., Sanchez M.G., El Hajj H., Milior G., Olmos-Alonso A., Gomez-Nicola D., Luheshi G., Vallieres L. (2016). Dark microglia: A new phenotype predominantly associated with pathological states. Glia.

[15] Nakanishi H., Wu Z. (2009). Microglia-aging: Roles of microglial lysosome- and mitochondria-derived reactive oxygen species in brain aging. Behav. Brain Res..

[16] Mrak R.E., Griffin S.T., Graham D.I. (1997). Aging-associated changes in human brain. J. Neuropathol. Exp. Neurol..

[17] Gray D.A., Woulfe J. (2005). Lipofuscin and aging: A matter of toxic waste. Sci. Aging Knowl. Environ..

[18] Ahmed Z., Sheng H., Xu Y.F., Lin W.L., Innes A.E., Gass J., Yu X., Wuertzer C.A., Hou H., Chiba S. (2010). Accelerated lipofuscinosis and ubiquitination in granulin knockout mice suggest a role for progranulin in successful aging. Am. J. Pathol..

[19] Ritzel R.M., Patel A.R., Pan S., Crapser J., Hammond M., Jellison E., McCullough L.D. (2015). Age- and location-related changes in microglial function. Neurobiol. Aging.

[20] Oliveira V.C., Carrara R.C., Simoes D.L., Saggioro F.P., Carlotti C.G., Covas D.T., Neder L. (2010). Sudan Black B treatment reduces autofluorescence and improves resolution of in situ hybridization specific fluorescent signals of brain sections. Histol. Histopathol..

[21] Moussaud S., Draheim H.J. (2010). A new method to isolate microglia from adult mice and culture them for an extended period of time. J. Neurosci. Methods.

[22] Von Bernhardi R., Tichauer J., Eugenin-von Bernhardi L. (2011). Proliferating culture of aged microglia for the study of neurodegenerative diseases. J. Neurosci. Methods.

[23] Caldeira C., Oliveira A.F., Cunha C., Vaz A.R., Falcao A.S., Fernandes A., Brites D. (2014). Microglia change from a reactive to an age-like phenotype with the time in culture. Front. Cell. Neurosci..

[24] Martinez F.O., Gordon S. (2014). The M1 and M2 paradigm of macrophage activation: Time for reassessment. F1000Prime Rep..

[25] Ransohoff R.M. (2016). A polarizing question: Do M1 and M2 microglia exist?. Nat. Neurosci..

[26] Lee D.C., Ruiz C.R., Lebson L., Selenica M.L., Rizer J., Hunt J.B., Rojiani R., Reid P., Kammath S., Nash K. (2013). Aging enhances classical activation but mitigates alternative activation in the central nervous system. Neurobiol. Aging.

[27] Tchkonia T., Zhu Y., van Deursen J., Campisi J., Kirkland J.L. (2013). Cellular senescence and the senescent secretory phenotype: Therapeutic opportunities. J. Clin. Investig..

[28] Coppe J.P., Patil C.K., Rodier F., Sun Y., Munoz D.P., Goldstein J., Nelson P.S., Desprez P.Y., Campisi J. (2008). Senescence-associated secretory phenotypes reveal cell-nonautonomous functions of oncogenic RAS and the p53 tumor suppressor. PLoS Biol..

[29] Brown D.I., Griendling K.K. (2015). Regulation of signal transduction by reactive oxygen species in the cardiovascular system. Circ. Res..

[30] Kang J., Park E.J., Jou I., Kim J.H., Joe E.H. (2001). Reactive oxygen species mediate Aβ(25–35)-induced activation of BV-2 microglia. Neuroreport.

[31] Qin L., Liu Y., Wang T., Wei S.J., Block M.L., Wilson B., Liu B., Hong J.S. (2004). NADPH oxidase mediates lipopolysaccharide-induced neurotoxicity and proinflammatory gene expression in activated microglia. J. Biol. Chem..

[32] Bordt E.A., Polster B.M. (2014). NADPH oxidase- and mitochondria-derived reactive oxygen species in proinflammatory microglial activation: A bipartisan affair?. Free Radic. Biol. Med..

[33] Ansari M.A., Scheff S.W. (2011). NADPH-oxidase activation and cognition in Alzheimer disease progression. Free Radic. Biol. Med..

[34] Lull M.E., Levesque S., Surace M.J., Block M.L. (2011). Chronic apocynin treatment attenuates β amyloid plaque size and microglial number in hAPP(751)(SL) mice. PLoS ONE.

[35] Han B.H., Zhou M.L., Johnson A.W., Singh I., Liao F., Vellimana A.K., Nelson J.W., Milner E., Cirrito J.R., Basak J. (2015). Contribution of reactive oxygen species to cerebral amyloid angiopathy, vasomotor dysfunction, and microhemorrhage in aged Tg2576 mice. Proc. Natl. Acad. Sci. USA.

[36] Qin L., Liu Y., Hong J.S., Crews F.T. (2013). NADPH oxidase and aging drive microglial activation, oxidative stress, and dopaminergic neurodegeneration following systemic LPS administration. Glia.

[37] Zhang B., Bailey W.M., McVicar A.L., Gensel J.C. (2016). Age increases reactive oxygen species production in macrophages and potentiates oxidative damage after spinal cord injury. Neurobiol. Aging.

[38] Njie E.G., Boelen E., Stassen F.R., Steinbusch H.W., Borchelt D.R., Streit W.J. (2012). Ex vivo cultures of microglia from young and aged rodent brain reveal age-related changes in microglial function. Neurobiol. Aging.

[39] Von Bernhardi R., Eugenin-von Bernhardi L., Eugenin J. (2015). Microglial cell dysregulation in brain aging and neurodegeneration. Front. Aging Neurosci..

[40] Wu Z., Yu J., Zhu A., Nakanishi H. (2016). Nutrients, Microglia Aging, and Brain Aging. Oxid. Med. Cell. Longev..

[41] Denieffe S., Kelly R.J., McDonald C., Lyons A., Lynch M.A. (2013). Classical activation of microglia in CD200-deficient mice is a consequence of blood brain barrier permeability and infiltration of peripheral cells. Brain Behav. Immun..

[42] Costello D.A., Lyons A., Denieffe S., Browne T.C., Cox F.F., Lynch M.A. (2011). Long term potentiation is impaired in membrane glycoprotein CD200-deficient mice: A role for Toll-like receptor activation. J. Biol. Chem..

[43] Ritzel R.M., Crapser J., Patel A.R., Verma R., Grenier J.M., Chauhan A., Jellison E.R., McCullough L.D. (2016). Age-Associated Resident Memory CD8 T Cells in the Central Nervous System Are Primed To Potentiate Inflammation after Ischemic Brain Injury. J. Immunol. (Baltimore, Md.: 1950).

[44] Shrivastava K., Gonzalez P., Acarin L. (2012). The immune inhibitory complex CD200/CD200R is developmentally regulated in the mouse brain. J. Comp. Neurol..

[45] Wang X.J., Zhang S., Yan Z.Q., Zhao Y.X., Zhou H.Y., Wang Y., Lu G.Q., Zhang J.D. (2011). Impaired CD200-CD200R-mediated microglia silencing enhances midbrain dopaminergic neurodegeneration: Roles of aging, superoxide, NADPH oxidase, and p38 MAPK. Free Radic. Biol. Med..

[46] Frank M.G., Barrientos R.M., Biedenkapp J.C., Rudy J.W., Watkins L.R., Maier S.F. (2006). mRNA up-regulation of MHC II and pivotal pro-inflammatory genes in normal brain aging. Neurobiol. Aging.

[47] Walker D.G., Dalsing-Hernandez J.E., Campbell N.A., Lue L.F. (2009). Decreased expression of CD200 and CD200 receptor in Alzheimer’s disease: A potential mechanism leading to chronic inflammation. Exp. Neurol..

[48] Lyons A., McQuillan K., Deighan B.F., O’Reilly J.A., Downer E.J., Murphy A.C., Watson M., Piazza A., O’Connell F., Griffin R. (2009). Decreased neuronal CD200 expression in IL-4-deficient mice results in increased neuroinflammation in response to lipopolysaccharide. Brain Behav. Immun..

[49] Cox F.F., Carney D., Miller A.M., Lynch M.A. (2012). CD200 fusion protein decreases microglial activation in the hippocampus of aged rats. Brain Behav. Immun..

[50] Lyons A., Downer E.J., Costello D.A., Murphy N., Lynch M.A. (2012). Dok2 mediates the CD200Fc attenuation of Abeta-induced changes in glia. J. Neuroinflamm..

[51] Varnum M.M., Kiyota T., Ingraham K.L., Ikezu S., Ikezu T. (2015). The anti-inflammatory glycoprotein, CD200, restores neurogenesis and enhances amyloid phagocytosis in a mouse model of Alzheimer’s disease. Neurobiol. Aging.

[52] Lyons A., Minogue A.M., Jones R.S., Fitzpatrick O., Noonan J., Campbell V.A., Lynch M.A. (2016). Analysis of the Impact of CD200 on Phagocytosis. Mol. Neurobiol..

[53] Zujovic V., Benavides J., Vige X., Carter C., Taupin V. (2000). Fractalkine modulates TNF-α secretion and neurotoxicity induced by microglial activation. Glia.

[54] Lyons A., Lynch A.M., Downer E.J., Hanley R., O’Sullivan J.B., Smith A., Lynch M.A. (2009). Fractalkine-induced activation of the phosphatidylinositol-3 kinase pathway attentuates microglial activation in vivo and in vitro. J. Neurochem..

[55] Fenn A.M., Smith K.M., Lovett-Racke A.E., Guerau-de-Arellano M., Whitacre C.C., Godbout J.P. (2013). Increased micro-RNA 29b in the aged brain correlates with the reduction of insulin-like growth factor-1 and fractalkine ligand. Neurobiol. Aging.

[56] Duan R.S., Yang X., Chen Z.G., Lu M.O., Morris C., Winblad B., Zhu J. (2008). Decreased fractalkine and increased IP-10 expression in aged brain of APP(swe) transgenic mice. Neurochem. Res..

[57] Cribbs D.H., Berchtold N.C., Perreau V., Coleman P.D., Rogers J., Tenner A.J., Cotman C.W. (2012). Extensive innate immune gene activation accompanies brain aging, increasing vulnerability to cognitive decline and neurodegeneration: A microarray study. J. Neuroinflamm..

[58] Bachstetter A.D., Morganti J.M., Jernberg J., Schlunk A., Mitchell S.H., Brewster K.W., Hudson C.E., Cole M.J., Harrison J.K., Bickford P.C. (2011). Fractalkine and CX 3 CR1 regulate hippocampal neurogenesis in adult and aged rats. Neurobiol. Aging.

[59] Wynne A.M., Henry C.J., Huang Y., Cleland A., Godbout J.P. (2010). Protracted downregulation of CX3CR1 on microglia of aged mice after lipopolysaccharide challenge. Brain Behav. Immun..

[60] Wu J., Bie B., Yang H., Xu J.J., Brown D.L., Naguib M. (2013). Suppression of central chemokine fractalkine receptor signaling alleviates amyloid-induced memory deficiency. Neurobiol. Aging.

[61] Lee S., Xu G., Jay T.R., Bhatta S., Kim K.W., Jung S., Landreth G.E., Ransohoff R.M., Lamb B.T. (2014). Opposing effects of membrane-anchored CX3CL1 on amyloid and tau pathologies via the p38 MAPK pathway. J. Neurosci..

[62] Lee S., Varvel N.H., Konerth M.E., Xu G., Cardona A.E., Ransohoff R.M., Lamb B.T. (2010). CX3CR1 deficiency alters microglial activation and reduces β-amyloid deposition in two Alzheimer’s disease mouse models. Am. J. Pathol..

[63] Cho S.H., Sun B., Zhou Y., Kauppinen T.M., Halabisky B., Wes P., Ransohoff R.M., Gan L. (2011). CX3CR1 protein signaling modulates microglial activation and protects against plaque-independent cognitive deficits in a mouse model of Alzheimer disease. J. Biol. Chem..

[64] Nash K.R., Lee D.C., Hunt J.B., Morganti J.M., Selenica M.L., Moran P., Reid P., Brownlow M., Guang-Yu Yang C., Savalia M. (2013). Fractalkine overexpression suppresses tau pathology in a mouse model of tauopathy. Neurobiol. Aging.

[65] Orre M., Kamphuis W., Osborn L.M., Jansen A.H., Kooijman L., Bossers K., Hol E.M. (2014). Isolation of glia from Alzheimer’s mice reveals inflammation and dysfunction. Neurobiol. Aging.

[66] Floden A.M., Combs C.K. (2011). Microglia demonstrate age-dependent interaction with amyloid-β fibrils. J. Alzheimer’s Dis. JAD.

[67] Lynch A.M., Murphy K.J., Deighan B.F., O’Reilly J.A., Gun’ko Y.K., Cowley T.R., Gonzalez-Reyes R.E., Lynch M.A. (2010). The impact of glial activation in the aging brain. Aging Dis..

[68] Hendrickx D.A., Schuurman K.G., van Draanen M., Hamann J., Huitinga I. (2014). Enhanced uptake of multiple sclerosis-derived myelin by THP-1 macrophages and primary human microglia. J. Neuroinflamm..

[69] Gitik M., Liraz-Zaltsman S., Oldenborg P.A., Reichert F., Rotshenker S. (2011). Myelin down-regulates myelin phagocytosis by microglia and macrophages through interactions between CD47 on myelin and SIRPα (signal regulatory protein-α) on phagocytes. J. Neuroinflamm..

[70] Combs C.K., Karlo J.C., Kao S.C., Landreth G.E. (2001). β-Amyloid stimulation of microglia and monocytes results in TNFα-dependent expression of inducible nitric oxide synthase and neuronal apoptosis. J. Neurosci..

[71] Bliederhaeuser C., Grozdanov V., Speidel A., Zondler L., Ruf W.P., Bayer H., Kiechle M., Feiler M.S., Freischmidt A., Brenner D. (2016). Age-dependent defects of α-synuclein oligomer uptake in microglia and monocytes. Acta. Neuropathol..

[72] Tichauer J.E., Flores B., Soler B., Eugenin-von Bernhardi L., Ramirez G., von Bernhardi R. (2014). Age-dependent changes on TGFbeta1 Smad3 pathway modify the pattern of microglial cell activation. Brain Behav. Immun..

[73] Chakrabarty P., Li A., Ceballos-Diaz C., Eddy J.A., Funk C.C., Moore B., DiNunno N., Rosario A.M., Cruz P.E., Verbeeck C. (2015). IL-10 alters immunoproteostasis in APP mice, increasing plaque burden and worsening cognitive behavior. Neuron.

[74] Guillot-Sestier M.V., Doty K.R., Gate D., Rodriguez J., Leung B.P., Rezai-Zadeh K., Town T. (2015). Il10 deficiency rebalances innate immunity to mitigate Alzheimer-like pathology. Neuron.

[75] Kan M.J., Lee J.E., Wilson J.G., Everhart A.L., Brown C.M., Hoofnagle A.N., Jansen M., Vitek M.P., Gunn M.D., Colton C.A. (2015). Arginine deprivation and immune suppression in a mouse model of Alzheimer’s disease. J. Neurosci..

[76] Dagher N.N., Najafi A.R., Kayala K.M., Elmore M.R., White T.E., Medeiros R., West B.L., Green K.N. (2015). Colony-stimulating factor 1 receptor inhibition prevents microglial plaque association and improves cognition in 3xTg-AD mice. J. Neuroinflamm..

[77] Rice R.A., Spangenberg E.E., Yamate-Morgan H., Lee R.J., Arora R.P., Hernandez M.X., Tenner A.J., West B.L., Green K.N. (2015). Elimination of Microglia Improves Functional Outcomes Following Extensive Neuronal Loss in the Hippocampus. J. Neurosci..

[78] Feng X., Jopson T.D., Paladini M.S., Liu S., West B.L., Gupta N., Rosi S. (2016). Colony-stimulating factor 1 receptor blockade prevents fractionated whole-brain irradiation-induced memory deficits. J. Neuroinflamm..

[79] Acharya M.M., Green K.N., Allen B.D., Najafi A.R., Syage A., Minasyan H., Le M.T., Kawashita T., Giedzinski E., Parihar V.K. (2016). Elimination of microglia improves cognitive function following cranial irradiation. Sci. Rep..

[80] Cunningham C. (2013). Microglia and neurodegeneration: The role of systemic inflammation. Glia.

[81] Baruch K., Deczkowska A., David E., Castellano J.M., Miller O., Kertser A., Berkutzki T., Barnett-Itzhaki Z., Bezalel D., Wyss-Coray T. (2014). Aging. Aging-induced type I interferon response at the choroid plexus negatively affects brain function. Science.

[82] Soto I., Graham L.C., Richter H.J., Simeone S.N., Radell J.E., Grabowska W., Funkhouser W.K., Howell M.C., Howell G.R. (2015). APOE Stabilization by Exercise Prevents Aging Neurovascular Dysfunction and Complement Induction. PLoS Biol..

[83] Montagne A., Barnes S.R., Sweeney M.D., Halliday M.R., Sagare A.P., Zhao Z., Toga A.W., Jacobs R.E., Liu C.Y., Amezcua L. (2015). Blood-brain barrier breakdown in the aging human hippocampus. Neuron.

[84] Grabert K., Michoel T., Karavolos M.H., Clohisey S., Baillie J.K., Stevens M.P., Freeman T.C., Summers K.M., McColl B.W. (2016). Microglial brain region-dependent diversity and selective regional sensitivities to aging. Nat. Neurosci..

[85] Villeda S.A., Luo J., Mosher K.I., Zou B., Britschgi M., Bieri G., Stan T.M., Fainberg N., Ding Z., Eggel A. (2011). The ageing systemic milieu negatively regulates neurogenesis and cognitive function. Nature.

[86] Smith L.K., He Y., Park J.S., Bieri G., Snethlage C.E., Lin K., Gontier G., Wabl R., Plambeck K.E., Udeochu J. (2015). beta2-microglobulin is a systemic pro-aging factor that impairs cognitive function and neurogenesis. Nat. Med..

[87] Carabotti M., Scirocco A., Maselli M.A., Severi C. (2015). The gut-brain axis: Interactions between enteric microbiota, central and enteric nervous systems. Ann. Gastroenterol..

[88] Erny D., Hrabe de Angelis A.L., Jaitin D., Wieghofer P., Staszewski O., David E., Keren-Shaul H., Mahlakoiv T., Jakobshagen K., Buch T. (2015). Host microbiota constantly control maturation and function of microglia in the CNS. Nat. Neurosci..

[89] Graham L.C., Harder J.M., Soto I., de Vries W.N., John S.W., Howell G.R. (2016). Chronic consumption of a western diet induces robust glial activation in aging mice and in a mouse model of Alzheimer’s disease. Sci. Rep..

[90] Kohman R.A., Bhattacharya T.K., Wojcik E., Rhodes J.S. (2013). Exercise reduces activation of microglia isolated from hippocampus and brain of aged mice. J. Neuroinflamm..

[91] Loncarevic-Vasiljkovic N., Pesic V., Todorovic S., Popic J., Smiljanic K., Milanovic D., Ruzdijic S., Kanazir S. (2012). Caloric restriction suppresses microglial activation and prevents neuroapoptosis following cortical injury in rats. PLoS ONE.

[92] D’Mello C., Ronaghan N., Zaheer R., Dicay M., Le T., MacNaughton W.K., Surrette M.G., Swain M.G. (2015). Probiotics Improve Inflammation-Associated Sickness Behavior by Altering Communication between the Peripheral Immune System and the Brain. J. Neurosci..

[93] Popa-Wagner A., Buga A.M., Tica A.A., Albu C.V. (2014). Perfusion deficits, inflammation and aging precipitate depressive behaviour. Biogerontology.

[94] Bickford P.C., Flowers A., Grimmig B. (2017). Aging leads to altered microglial function that reduces brain resiliency increasing vulnerability to neurodegenerative diseases. Exp. Gerontol..

[95] Blazer D.G., Yaffe K., Karlawish J. (2015). Cognitive aging: A report from the Institute of Medicine. JAMA.

[96] Patterson S.L. (2015). Immune dysregulation and cognitive vulnerability in the aging brain: Interactions of microglia, IL-1beta, BDNF and synaptic plasticity. Neuropharmacology.

[97] Androsova G., Krause R., Winterer G., Schneider R. (2015). Biomarkers of postoperative delirium and cognitive dysfunction. Front. Aging Neurosci..

[98] Gleason L.J., Schmitt E.M., Kosar C.M., Tabloski P., Saczynski J.S., Robinson T., Cooper Z., Rogers S.O., Jones R.N., Marcantonio E.R. (2015). Effect of Delirium and Other Major Complications on Outcomes After Elective Surgery in Older Adults. JAMA Surg..

[99] Inouye S.K., Westendorp R.G., Saczynski J.S. (2014). Delirium in elderly people. Lancet.

[100] Vasunilashorn S.M., Ngo L., Inouye S.K., Libermann T.A., Jones R.N., Alsop D.C., Guess J., Jastrzebski S., McElhaney J.E., Kuchel G.A. (2015). Cytokines and Postoperative Delirium in Older Patients Undergoing Major Elective Surgery. J. Gerontol. Ser. A Biol. Sci. Med. Sci..

[101] Godbout J.P., Chen J., Abraham J., Richwine A.F., Berg B.M., Kelley K.W., Johnson R.W. (2005). Exaggerated neuroinflammation and sickness behavior in aged mice following activation of the peripheral innate immune system. FASEB J. Off. Publ. Fed. Am. Soc. Exp. Biol..

[102] Godbout J.P., Moreau M., Lestage J., Chen J., Sparkman N.L., O’Connor J., Castanon N., Kelley K.W., Dantzer R., Johnson R.W. (2008). Aging exacerbates depressive-like behavior in mice in response to activation of the peripheral innate immune system. Neuropsychopharmacol. Off. Publ. Am. Coll. Neuropsychopharmacol..

[103] Simone M.J., Tan Z.S. (2011). The role of inflammation in the pathogenesis of delirium and dementia in older adults: A review. CNS Neurosci. Ther..

[104] Fonken L.K., Frank M.G. (2016). The Alarmin HMGB1 Mediates Age-Induced Neuroinflammatory Priming. J. Neurosci..

[105] Huang Y., Henry C.J., Dantzer R., Johnson R.W., Godbout J.P. (2008). Exaggerated sickness behavior and brain proinflammatory cytokine expression in aged mice in response to intracerebroventricular lipopolysaccharide. Neurobiol. Aging.

[106] Rosczyk H.A., Sparkman N.L., Johnson R.W. (2008). Neuroinflammation and cognitive function in aged mice following minor surgery. Exp. Gerontol..

[107] Peng M., Zhang C., Dong Y., Zhang Y., Nakazawa H., Kaneki M., Zheng H., Shen Y., Marcantonio E.R., Xie Z. (2016). Battery of behavioral tests in mice to study postoperative delirium. Sci. Rep..

[108] Ren Q., Peng M., Dong Y., Zhang Y., Chen M., Yin N., Marcantonio E.R., Xie Z. (2015). Surgery plus anesthesia induces loss of attention in mice. Front. Cell. Neurosci..

[109] Buchanan J.B., Sparkman N.L., Chen J., Johnson R.W. (2008). Cognitive and neuroinflammatory consequences of mild repeated stress are exacerbated in aged mice. Psychoneuroendocrinology.

[110] Dantzer R., Capuron L., Irwin M.R., Miller A.H., Ollat H., Perry V.H., Rousey S., Yirmiya R. (2008). Identification and treatment of symptoms associated with inflammation in medically ill patients. Psychoneuroendocrinology.

[111] Coronado V.G., Thomas K.E., Sattin R.W., Johnson R.L. (2005). The CDC traumatic brain injury surveillance system: Characteristics of persons aged 65 years and older hospitalized with a TBI. J. Head Trauma Rehabil..

[112] Faul M., Coronado V. (2015). Epidemiology of traumatic brain injury. Handb. Clin. Neurol..

[113] Mosenthal A.C., Lavery R.F., Addis M., Kaul S., Ross S., Marburger R., Deitch E.A., Livingston D.H. (2002). Isolated traumatic brain injury: Age is an independent predictor of mortality and early outcome. J. Trauma.

[114] Stocchetti N., Paterno R., Citerio G., Beretta L., Colombo A. (2012). Traumatic brain injury in an aging population. J. Neurotrauma.

[115] Livingston D.H., Lavery R.F., Mosenthal A.C., Knudson M.M., Lee S., Morabito D., Manley G.T., Nathens A., Jurkovich G., Hoyt D.B. (2005). Recovery at one year following isolated traumatic brain injury: A Western Trauma Association prospective multicenter trial. J. Trauma.

[116] Testa J.A., Malec J.F., Moessner A.M., Brown A.W. (2005). Outcome after traumatic brain injury: Effects of aging on recovery. Arch. Phys. Med. Rehabil..

[117] Gardner R.C., Burke J.F., Nettiksimmons J., Kaup A., Barnes D.E., Yaffe K. (2014). Dementia risk after traumatic brain injury vs nonbrain trauma: The role of age and severity. JAMA Neurol..

[118] Washington P.M., Morffy N., Parsadanian M., Zapple D.N., Burns M.P. (2014). Experimental traumatic brain injury induces rapid aggregation and oligomerization of amyloid-beta in an Alzheimer’s disease mouse model. J. Neurotrauma.

[119] Chiu C.C., Liao Y.E., Yang L.Y., Wang J.Y., Tweedie D., Karnati H.K., Greig N.H., Wang J.Y. (2016). Neuroinflammation in animal models of traumatic brain injury. J. Neurosci. Methods.

[120] Johnson V.E., Meaney D.F., Cullen D.K., Smith D.H. (2015). Animal models of traumatic brain injury. Handb. Clin. Neurol..

[121] Xiong Y., Mahmood A., Chopp M. (2013). Animal models of traumatic brain injury. Nat. Rev. Neurosci..

[122] Kumar A., Stoica B.A., Sabirzhanov B., Burns M.P., Faden A.I., Loane D.J. (2013). Traumatic brain injury in aged animals increases lesion size and chronically alters microglial/macrophage classical and alternative activation states. Neurobiol. Aging.

[123] Fournier A.E., Takizawa B.T., Strittmatter S.M. (2003). Rho kinase inhibition enhances axonal regeneration in the injured CNS. J. Neurosci. Off. J. Soc. Neurosci..

[124] Liu G., Ni J., Mao L., Yan M., Pang T., Liao H. (2015). Expression of Nogo receptor 1 in microglia during development and following traumatic brain injury. Brain Res..

[125] Sandu R.E., Buga A.M., Balseanu A.T., Moldovan M., Popa-Wagner A. (2016). Twenty-four hours hypothermia has temporary efficacy in reducing brain infarction and inflammation in aged rats. Neurobiol. Aging.

[126] Sandu R.E., Uzoni A., Ciobanu O., Moldovan M., Anghel A., Radu E., Coogan A.N., Popa-Wagner A. (2016). Post-stroke gaseous hypothermia increases vascular density but not neurogenesis in the ischemic penumbra of aged rats. Restor. Neurol. Neurosci..

[127] Buga A.M., Di Napoli M., Popa-Wagner A. (2013). Preclinical models of stroke in aged animals with or without comorbidities: Role of neuroinflammation. Biogerontology.

[128] Crapser J., Ritzel R., Verma R., Venna V.R., Liu F., Chauhan A., Koellhoffer E., Patel A., Ricker A., Maas K. (2016). Ischemic stroke induces gut permeability and enhances bacterial translocation leading to sepsis in aged mice. Aging.

[129] Sieber M.W., Claus R.A., Witte O.W., Frahm C. (2011). Attenuated inflammatory response in aged mice brains following stroke. PLoS ONE.

[130] Shin J.A., Jeong S.I., Kim M., Yoon J.C., Kim H.S., Park E.M. (2015). Visceral adipose tissue inflammation is associated with age-related brain changes and ischemic brain damage in aged mice. Brain Behav. Immun..

[131] Hu X., Li P., Guo Y., Wang H., Leak R.K., Chen S., Gao Y., Chen J. (2012). Microglia/macrophage polarization dynamics reveal novel mechanism of injury expansion after focal cerebral ischemia. Stroke.

[132] Suenaga J., Hu X., Pu H., Shi Y., Hassan S.H., Xu M., Leak R.K., Stetler R.A., Gao Y., Chen J. (2015). White matter injury and microglia/macrophage polarization are strongly linked with age-related long-term deficits in neurological function after stroke. Exp. Neurol..

[133] Moraga A., Pradillo J.M., Garcia-Culebras A., Palma-Tortosa S., Ballesteros I., Hernandez-Jimenez M., Moro M.A., Lizasoain I. (2015). Aging increases microglial proliferation, delays cell migration, and decreases cortical neurogenesis after focal cerebral ischemia. J. Neuroinflamm..

[134] Salloway S., Sperling R., Fox N.C., Blennow K., Klunk W., Raskind M., Sabbagh M., Honig L.S., Porsteinsson A.P., Ferris S. (2014). Two phase 3 trials of bapineuzumab in mild-to-moderate Alzheimer’s disease. N. Engl. J. Med..

[135] Brody M., Liu E., Di J., Lu M., Margolin R.A., Werth J.L., Booth K., Shadman A., Brashear H.R., Novak G. (2016). A Phase II, Randomized, Double-Blind, Placebo-Controlled Study of Safety, Pharmacokinetics, and Biomarker Results of Subcutaneous Bapineuzumab in Patients with mild to moderate Alzheimer’s disease. J. Alzheimer’s Dis. JAD.

[136] Ivanoiu A., Pariente J., Booth K., Lobello K., Luscan G., Hua L., Lucas P., Styren S., Yang L., Li D. (2016). Long-term safety and tolerability of bapineuzumab in patients with Alzheimer’s disease in two phase 3 extension studies. Alzheimer’s Res. Ther..

[137] Vandenberghe R., Rinne J.O., Boada M., Katayama S., Scheltens P., Vellas B., Tuchman M., Gass A., Fiebach J.B., Hill D. (2016). Bapineuzumab for mild to moderate Alzheimer’s disease in two global, randomized, phase 3 trials. Alzheimer’s Res. Ther..

[138] Sevigny J., Chiao P., Bussiere T., Weinreb P.H., Williams L., Maier M., Dunstan R., Salloway S., Chen T., Ling Y. (2016). The antibody aducanumab reduces Aβ plaques in Alzheimer’s disease. Nature.

[139] Querfurth H.W., La Ferla F.M. (2010). Alzheimer’s disease. N. Engl. J. Med..

[140] Ries M., Sastre M. (2016). Mechanisms of Abeta Clearance and Degradation by Glial Cells. Front. Aging Neurosci..

[141] Rodriguez J.J., Butt A.M., Gardenal E., Parpura V., Verkhratsky A. (2016). Complex and differential glial responses in Alzheimer’s disease and ageing. Curr. Alzheimer Res..

[142] Udeochu J.C., Shea J.M., Villeda S.A. (2016). Microglia communication: Parallels between aging and Alzheimer’s disease. Clin. Exp. Neuroimmunol..

[143] Braak H., Braak E. (1997). Frequency of stages of Alzheimer-related lesions in different age categories. Neurobiol. Aging.

[144] Peters F., Collette F., Degueldre C., Sterpenich V., Majerus S., Salmon E. (2009). The neural correlates of verbal short-term memory in Alzheimer’s disease: An fMRI study. Brain J. Neurol..

[145] Halle A., Hornung V., Petzold G.C., Stewart C.R., Monks B.G., Reinheckel T., Fitzgerald K.A., Latz E., Moore K.J., Golenbock D.T. (2008). The NALP3 inflammasome is involved in the innate immune response to amyloid-beta. Nat. Immunol..

[146] Heneka M.T., Kummer M.P., Stutz A., Delekate A., Schwartz S., Vieira-Saecker A., Griep A., Axt D., Remus A., Tzeng T.C. (2013). NLRP3 is activated in Alzheimer’s disease and contributes to pathology in APP/PS1 mice. Nature.

[147] Meda L., Cassatella M.A., Szendrei G.I., Otvos L., Baron P., Villalba M., Ferrari D., Rossi F. (1995). Activation of microglial cells by beta-amyloid protein and interferon-gamma. Nature.

[148] Zhao W., Zhang J., Davis E.G., Rebeck G.W. (2014). Aging reduces glial uptake and promotes extracellular accumulation of Abeta from a lentiviral vector. Front. Aging Neurosci..

[149] Hickman S.E., Allison E.K., El Khoury J. (2008). Microglial dysfunction and defective beta-amyloid clearance pathways in aging Alzheimer’s disease mice. J. Neurosci. Off. J. Soc. Neurosci..

[150] Middeldorp J., Lehallier B., Villeda S.A., Miedema S.S., Evans E., Czirr E., Zhang H., Luo J., Stan T., Mosher K.I. (2016). Preclinical Assessment of Young Blood Plasma for Alzheimer Disease. JAMA Neurol..

[151] Xiang Y., Bu X.L., Liu Y.H., Zhu C., Shen L.L., Jiao S.S., Zhu X.Y., Giunta B., Tan J., Song W.H. (2015). Physiological amyloid-beta clearance in the periphery and its therapeutic potential for Alzheimer’s disease. Acta Neuropathol..

[152] Wang Y., Ulland T.K., Ulrich J.D., Song W., Tzaferis J.A., Hole J.T., Yuan P., Mahan T.E., Shi Y., Gilfillan S. (2016). TREM2-mediated early microglial response limits diffusion and toxicity of amyloid plaques. J. Exp. Med..

[153] Gottfried E., Kunz-Schughart L.A., Weber A., Rehli M., Peuker A., Muller A., Kastenberger M., Brockhoff G., Andreesen R., Kreutz M. (2008). Expression of CD68 in non-myeloid cell types. Scand. J. Immunol..

